# Identification of Schwann Cells in Human Intracranial Arteries: Potential Regulatory Role in Atherosclerotic Plaque Progression

**DOI:** 10.1002/advs.202503033

**Published:** 2025-06-26

**Authors:** Zijue Wang, Yangzhige He, Weizhuang Yuan, Yijun Xia, Manqiu Ding, Zhen Chen, Naili Wang, Chao Ma, Xiaoyue Wang, Yan Xu, Weihai Xu

**Affiliations:** ^1^ Department of Neurology State Key Laboratory of Complex, Severe, and Rare Diseases Peking Union Medical College Hospital Chinese Academy of Medical Sciences and Peking Union Medical College Beijing 100730 China; ^2^ Center for bioinformatics National Infrastructures for Translational Medicine Institute of Clinical Medicine and Peking Union Medical College Hospital Chinese Academy of Medical Sciences and Peking Union Medical College Beijing China; ^3^ Department of Neurology and Stroke Center First Affiliated Hospital Sun Yat‐Sen University Guangzhou China; ^4^ Department of Plastic Surgery Peking Union Medical College Hospital Chinese Academy of Medical Sciences and Peking Union Medical College Beijing China; ^5^ National Human Brain Bank for Development and Function Department of Human Anatomy Histology and Embryology Institute of Basic Medical Sciences Chinese Academy of Medical Sciences Peking Union Medical Collage Beijing China

**Keywords:** autopsy, intracranial atherosclerosis, myelinated nerve fibers, SPP1‐ITGB1 signaling pathway, Schwann cells

## Abstract

Intracranial atherosclerosis (ICAS), a common cause of ischemic stroke, remains a therapeutic challenge due to complex intracranial anatomy and intervention risks. Although ICAS develops intracranially, cerebral artery innervation originates in the peripheral nervous system. The scarcity of human intracranial specimens has hindered investigations into the potential role of Schwann cells (SCs) in neurovascular homeostasis. Using multimodal analysis of plaque‐bearing and non‐plaque‐bearing intracranial artery samples from the same postmortem individuals (n = 16 donors), SCs associated with myelinated neural structures are identified. Quantitative ultrastructural evaluation reveals a 4.3‐fold increase in SC‐derived myelin sheaths within plaque‐bearing vessels (*P* <0.001). Single‐cell RNA sequencing (scRNA‐seq) of SCs demonstrates significant upregulation of genes involved in axonogenesis, axon ensheathment, axon guidance, synaptic transmission, and synaptic integration. Cell–cell communication analysis shows enhanced interactions between SCs and vascular smooth muscle cells (VSMCs) in plaque‐bearing vessels. Synaptic‐like structures are observed in the walls of intracranial arteries, along with a 2.9‐fold increase in VSMC‐innervating myelinated fibers (*P* <0.001). Ligand–receptor analysis indicates SPP1–ITGB1 signaling as a potential mediator of SCs‐VSMCs crosstalk. This study provides evidence for the involvement of SCs in ICAS pathobiology and proposes novel neurovascular targets for precision therapies in cerebrovascular disease.

## Graphical abstract

Schwann cells may facilitate intracranial plaque progression via neurovascular remodelingcting with vascular smooth muscle cells in atherosclerotic plaques, with the SPP1 ‐ ITGB1 signaling axis mediating this interaction as a novel mechanism.

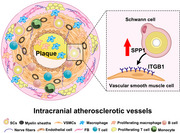

## 1 Introduction

Intracranial atherosclerosis (ICAS) is a common cause of ischemic stroke and a significant contributor to global morbidity and mortality.^[^ ^1^, ^2^ ^]^ Despite its clinical importance, the precise pathological mechanisms underlying ICAS remain poorly understood, posing a major challenge to the development of effective therapies.^[^ ^3^ ^]^ The unique structural characteristics of human intracranial arteries—such as their thin adventitia, sparse elastic fibers, absence of an external elastic lamina, and dense internal elastic lamina—suggest that the mechanisms driving atherosclerotic plaque formation in these vessels may differ from those in other vascular territories.^[^ ^4^ ^]^ Unlike coronary or carotid atherosclerosis, ICAS is less frequently treated surgically due to the anatomical complexity and heightened risk of intracranial interventions. This scarcity of surgical specimens limits in‐depth investigation into the specific molecular and cellular processes involved in intracranial plaque formation and progression. While considerable research has focused on endothelial dysfunction,^[^ ^5^, ^6^ ^]^ inflammatory responses,^[^ ^7^, ^8^ ^]^ and vascular smooth muscle cells (VSMCs) proliferation^[^ ^9^, ^10^ ^]^ in atherosclerosis, the role of other potential cellular contributors remains underexplored. Recent advances in vascular biology have begun to highlight the importance of previously overlooked cell types,^[^ ^11^, ^12^ ^]^ which may offer fresh insights into the pathophysiological mechanisms of ICAS.

Schwann cells (SCs) are known for their critical roles in myelination and nerve repair within the peripheral nervous system. They help maintain the structural and functional integrity of peripheral nerves, support axonal regeneration following injury, and modulate immune responses.^[^ ^13^, ^14^, ^15^, ^16^, ^17^ ^]^ However, the presence and function of myelinated nerve fibers in intracranial arterial vessels have been rarely studied,^[^ ^18^ ^]^ and it remains unclear whether and how these fibers contribute to vascular regulation and function in this unique anatomical context. Notably, recent studies have shown that oligodendrocytes—the central nervous system counterparts of SCs—accompany and regulate blood vessel development during brain maturation.^[^ ^11^, ^12^ ^]^ This raises the intriguing possibility that SCs may also play a role in the pathogenesis of intracranial vascular diseases, including ICAS. Given the distinct anatomical and physiological features of the intracranial vasculature, investigating whether SCs contribute to local vascular homeostasis and disease progression is both timely and warranted.

In this study, we focused on the involvement of SCs in forming atherosclerotic plaques by examining their presence in the walls of human intracranial arteries. Using transmission electron microscopy (TEM), we identified SCs in the arterial media, characterized by thin lamellar structures. Comparative analysis revealed increased clustering of SC‐derived myelin in plaque‐bearing vessels, suggesting a potential role for these cells in disease progression. To gain deeper insight into SC function, we employed single‐cell RNA sequencing (scRNA‐seq), which revealed upregulation of genes associated with axon ensheathment, axonogenesis, axon extension, axon guidance, synaptic rearrangement, synaptic transmission, and synaptic integration. Additionally, we observed enhanced interactions between SCs and VSMCs in plaque‐bearing vessels. Immunofluorescence (IF) staining demonstrated increased myelinated nerve fibers innervating VSMCs in these vessels. Further analysis indicated that SCs communicate with VSMCs via the SPP1 (osteopontin)–ITGB1 signaling pathway, which may regulate plaque progression. This novel finding suggests a potential mechanism by which SCs could influence vascular remodeling in atherosclerosis.

This study identified SCs in the intracranial arterial wall through multimodal analysis and provided evidence of their potential involvement in the progression of ICAS. Understanding the role of SCs in ICAS pathogenesis forms the basis for identifying new therapeutic targets. Targeting the SPP1–ITGB1 signaling axis may modulate SC activity and their interactions with VSMCs, offering a promising treatment approach for patients with intracranial atherosclerotic stenosis.

## 2 Results

### 2.1 SCs are Identified in the Vascular Wall of Human Intracranial Arteries

**Figure 1:**
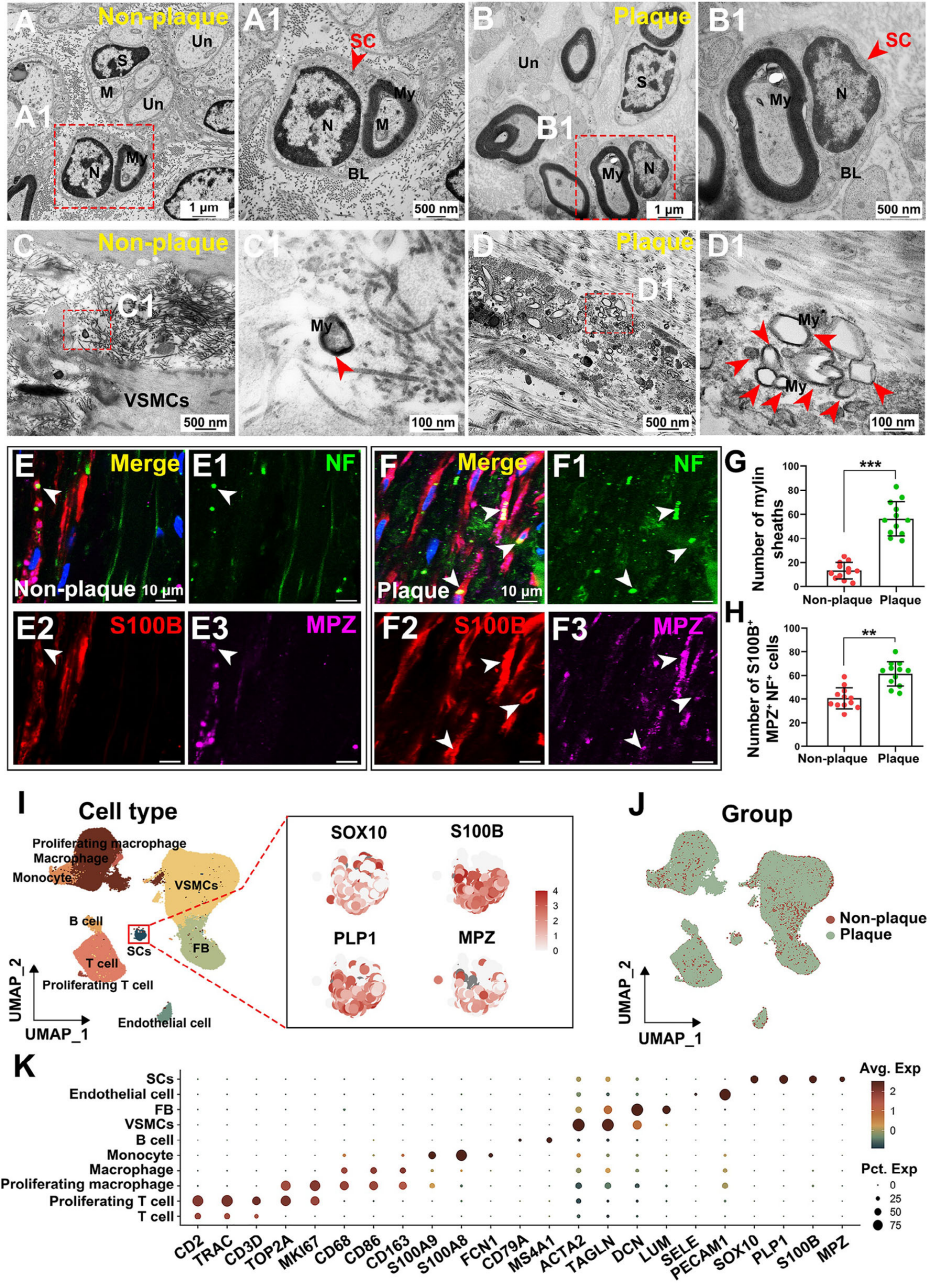
Identification of Schwann cells (SCs) in human intracranial arterial walls. A,B) Transmission electron microscopy (TEM) showed SCs (red arrowheads) and associated myelin sheaths in human intracranial arteries. Representative TEM image of SCs in non‐plaque‐bearing and plaque‐bearing vessels (A1, B1). Both myelinated (My) and unmyelinated (Un) nerve fibers were observed. They have many neurofilaments and a few mitochondria (M). Nucleus (N) seen in SCs. SCs are enveloped by a continuous basal lamina (BL). Scale bar, 1 µm and 500 nm. C,C1) Non‐plaque‐bearing vessels showed sparse myelinated structures (scale bar, 500 and 100 nm). D,D1) Plaque‐bearing vessels showed increased clustering of myelin sheaths compared to non‐plaque‐bearing vessels (scale bars, 500 and 100 nm). E,F) Immunofluorescence (IF) staining of myelinated SCs (S100B⁺/MPZ⁺/NF⁺, red/green/purple) in (E‐E3) non‐plaque‐bearing vessels and (F‐F3) plaque‐bearing vessels. SCs (S100B^+^) processes overlap with the axon (NF^+^) and myelin sheath (MPZ^+^) (white arrowheads, scale bar, 10 µm). G) Quantification of myelin sheaths in non‐plaque‐bearing vessels versus plaque‐bearing vessels. Data are presented as mean ± SD, *** *P* <0.001 (paired samples t‐test, n = 4 donors). H) Number of S100B⁺/MPZ⁺/NF^+^ cells in non‐plaque versus plaque‐bearing vessels. Data are presented as mean ± SD, ** *P* <0.01 (paired samples t‐test, n = 4 donors). I) UMAP plot showed clustering of 96034 cells into ten distinct populations, including SCs, VSMCs, endothelial cell, fibroblast (FB), B cell, monocyte, T cell, proliferating T cell, macrophage, and proliferating macrophage (n = 8 donors). UMAP plots showed specific marker expression for SCs, such as SOX10, S100B, PLP1, and MPZ. J) UMAP projection of cells from non‐plaque‐bearing and plaque‐bearing vessels. K) Dot plot illustrating the expression of key marker genes across cell types.

Oil Red O staining confirmed substantial lipid accumulation in plaque‐bearing vessels, in contrast to minimal staining in non‐plaque‐bearing vessels (Figure S1A,B, Supporting Information), validating the presence of intracranial atherosclerotic pathology. We identified SCs in the walls of human intracranial arteries (4 plaque‐bearing vessels vs 4 non‐plaque‐bearing vessels, n  = 4 donors) using TEM (**Figure** **1**A,B). SCs were located in the vascular wall, with scattered thin lamellar myelin structures observed in the arterial media (Figure 1C,D; Figure S1C–H, Supporting Information). Comparative analysis revealed a statistically significant increase in the number of myelin sheaths in plaque‐bearing vessels (Figure 1G). These sheaths were more densely clustered in plaque‐bearing vessels (Figure 1D; Figure S1D,F,H, Supporting Information). To confirm the identity of these SCs, we performed IF staining (4 plaque‐bearing vessels vs 4 non‐plaque‐bearing vessels, n  = 4 donors). The results showed a significant increase in the number of myelinating SCs (S100B^+^/MPZ^+^/NF^+^) in plaque‐bearing vessels compared to non‐plaque‐bearing vessels (Figure 1E,F,H). These findings suggest that SCs may contribute to the pathogenesis of ICAS, potentially playing a role in plaque formation and progression.

To further investigate the biological role of SCs in ICAS, we performed scRNA‐seq on arterial samples (n  = 8 donors). Unbiased clustering and Uniform Manifold Approximation and Projection (UMAP) analysis of 96 034 cells identified several distinct populations based on well‐established marker genes and cluster‐specific gene expression profiles. These included SCs, VSMCs, endothelial cell, fibroblast (FB), B cell, monocyte, T cell, proliferating T cell, macrophage, and proliferating macrophage (Figure 1I; Figure S2A, Supporting Information). The UMAP plot visualized the distribution of these cell types in plaque‐bearing versus non‐plaque‐bearing vessels (Figure 1J). Marker gene analysis further validated the identity of each cluster (Figure 1K), with SCs expressing well‐established markers such as SOX10, S100B, PLP1, and MPZ (Figure 1I). The paired dot plot showed the fraction of SCs in plaque‐bearing and non‐plaque‐bearing vessels for each donor (Figure S2B, Supporting Information). These results further supported the presence of SCs population in the vascular walls of human intracranial arteries.

### 2.2 SCs in Plaque‐Bearing Vessels Exhibit Increased Axon‐Related Genes and Myelinated Nerve Fibers

**Figure 2:**
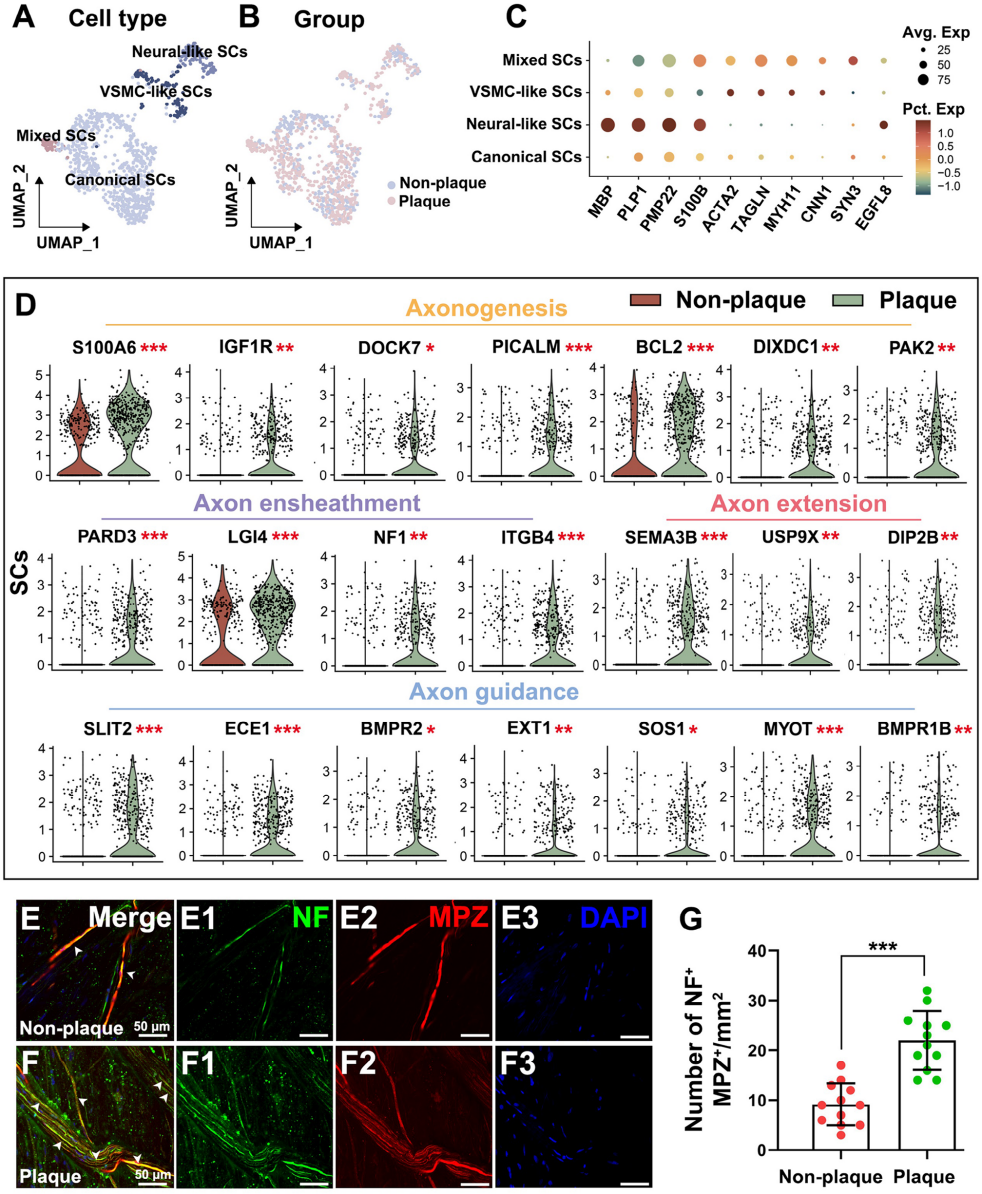
SCs neural remodeling in human intracranial arteries. A) UMAP plot showing SCs clustering of four distinct populations. B) UMAP projection of SCs from non‐plaque‐bearing and plaque‐bearing vessels. C) Dot plot showing the average expression and percentage of SC‐specific genes across different cell clusters. D) Violin plots reveal significant upregulation of SCs‐associated genes in plaque‐bearing vessels compared with non‐plaque‐bearing controls. These genes are involved in axonogenesis (S100A6, IGF1R, DOCK7, PICALM, BCL2, DIXDC1, PAK2), axon ensheathment (PARD3, LGI4, NF1, ITGB4), axon extension (SEMA3B, USP9X, DIP2B), and axon guidance (SLIT2, ECE1, BMPR2, EXT1, SOS1, MYOT and BMPR1B). *** *P* <0.001, ** *P* <0.01, and * *P* <0.05. n = 8 donors. E,F) IF staining showed myelinated nerve fibers (NF^+^ (green) and MPZ^+^ (red)) were increased in plaque‐bearing vessels (F) compared to non‐plaque‐bearing vessels (E). DAPI staining (blue) marks cell nuclei. Scale bars, 50 µm. G) Quantification of NF^+^/MPZ^+^ fibers per mm^2^ showed a significant increase in plaque‐bearing vessels compared to non‐plaque‐bearing vessels. Data are presented as mean ± SD, *** *P* <0.001 (paired samples t‐test, n = 4 donors).

To explore the functional heterogeneity of SCs, we performed scRNA‐seq and classified SCs subpopulations using dimensionality reduction and clustering. This analysis identified four distinct SC clusters—neural‐like SCs, VSMC‐like SCs, mixed SCs, and canonical SCs—across plaque‐bearing and non‐plaque‐bearing vessels (**Figure** **2**A,B). Marker gene expression analysis further validated the identity of these clusters (Figure 2C), indicating that SCs are not a homogeneous population. DoubletFinder analysis identified 30 doublets among 936 SCs (Figure S3J, Supporting Information), suggesting that most cells represent true biological populations.

To further assess SC subpopulation identities, we examined expression of established oligodendrocyte markers (OLIG2, MOG, MAG), and found no distinct clusters expressing these genes (Figure S2C, Supporting Information), confirming the absence of oligodendrocytes. While both myelinating and non‐myelinating SCs were detected, their clustering was insufficiently distinct to resolve them as separate groups (Figure S2C, Supporting Information).

To gain additional insight into the regulatory functions of SCs in intracranial arterial vessels, we compared differentially expressed genes (DEGs) between SCs from plaque‐bearing and non‐plaque‐bearing vessels. SCs from plaque‐bearing vessels exhibited increased expression of genes involved in axonogenesis, axon ensheathment, axon extension, and axon guidance—key structural processes required for long‐range signal conduction (Figure 2D). Gene set enrichment analysis (GSEA) revealed upregulation of axon guidance and axon development pathways in SCs from plaque‐bearing vessels (Figure S2D, Supporting Information). These findings suggest that SCs in plaque‐bearing regions may promote axonal growth and myelination.

IF staining further confirmed an increased presence of myelinated nerve fibers (NF^+^/MPZ^+^) in plaque‐bearing vessels compared to non‐plaque‐bearing vessels (Figure 2E,F). Statistical analysis showed a significant increase in the number of myelinated fibers in plaque‐bearing regions (Figure 2G), indicating enhanced innervation within atherosclerotic areas of intracranial arteries.

Collectively, these findings suggest that SCs may play a regulatory role in neurovascular remodeling during the progression of ICAS.

### 2.3 Enhanced Neurovascular Communication in Plaque‐Bearing Vessels

**Figure 3:**
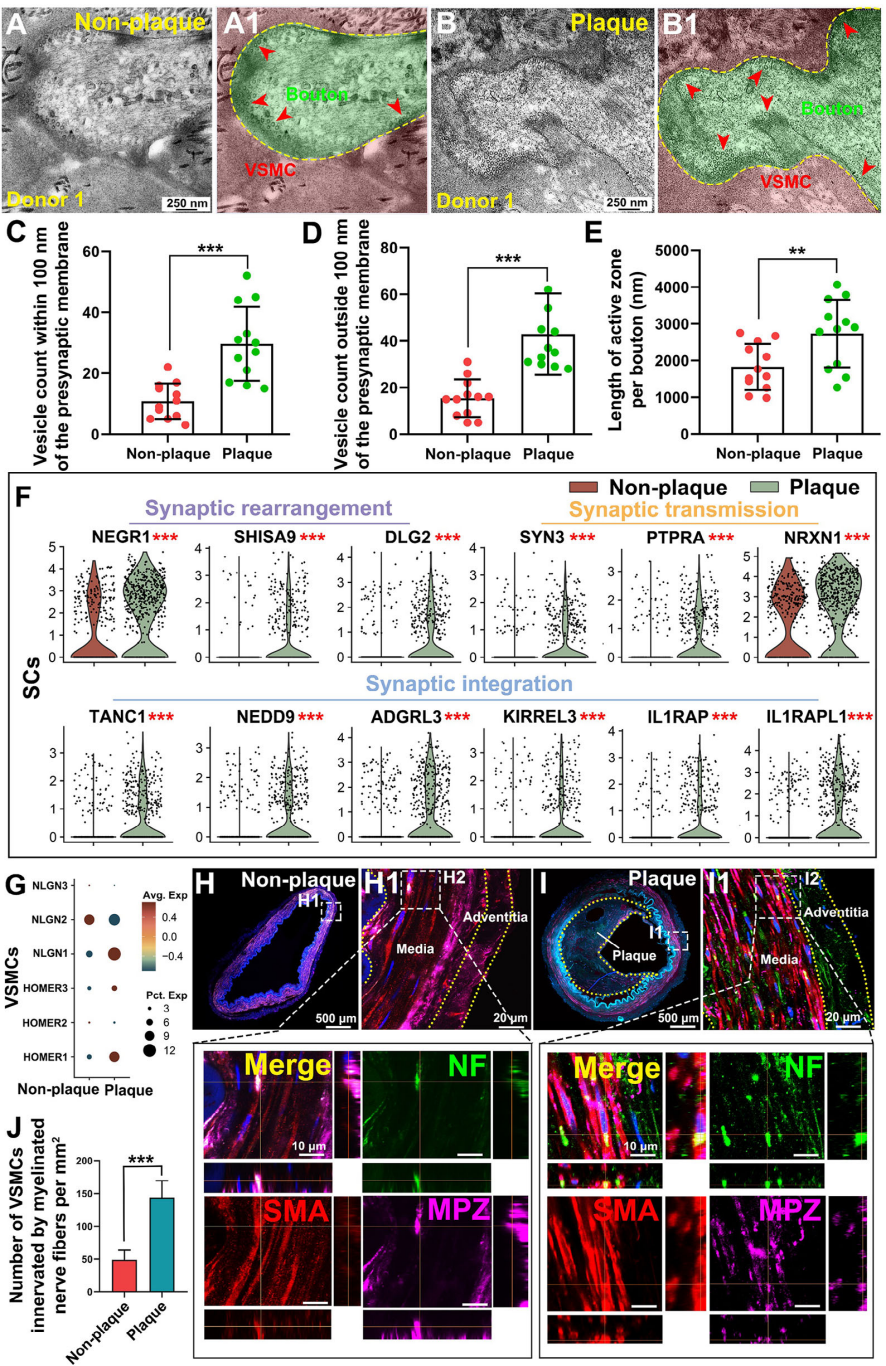
Synapse‐like structures increased in plaque‐bearing vessels. A,B) TEM showed synapse‐like structures between axonal terminals and VSMCs in non‐plaque‐bearing vessels (A, A1) and plaque‐bearing vessels (B, B1). Red arrowheads pointing to synaptic vesicles at the presynaptic membrane (scale bar, 250 nm, n = 4 donors). C) Plaque‐bearing vessels showed a significantly higher vesicle count near the presynaptic membrane (<100 nm) compared with the non‐plaque‐bearing vessels. Data are presented as mean ± SD, *** *P* <0.001 (paired samples t‐test). D) Plaque‐bearing vessels exhibited a significantly increased vesicle count further away from the presynaptic membrane (>100 nm). Data are presented as mean ± SD, *** *P* <0.001 (paired samples t‐test). E) Active zone length in the plaque‐bearing vessels was significantly longer than in the non‐plaque‐bearing vessels. Data are presented as mean ± SD, ** *P* <0.01 (paired samples t‐test). F) DEGs analysis between SCs in plaque‐bearing vessels and non‐plaque vessels showed upregulation of genes associated with synaptic rearrangement (NEGR1, SHISA9, and DLG2), synaptic transmission (SYN3, PTPRA, and NRXN1), and synaptic integration (TANC1, NEDD9, ADGRL3, KIRREL3, IL1RAP, and IL1RAPL). G) ScRNA‐seq revealed an increase in postsynaptic integration genes (HOMER1, HOMER3, and NLGN1) in VSMCs in the plaque‐bearing vessels (*** *P* < 0.001, n = 8 donors). H,I) IF staining showed an increased number of myelinated fibers (NF^+^/MPZ^+^) innervating VSMCs (SMA) in the plaque‐bearing vessels compared with non‐plaque‐bearing vessels (scale bars, 500, 20, and 10 µm). J) Quantitative results showed that the number of myelinated fibers in plaque‐bearing vessels was significantly higher than that in non‐plaque‐bearing vessels. Data are presented as mean ± SD, *** *P* <0.001 (paired samples t‐test, n = 4 donors).

To investigate the regulatory mechanisms underlying the upregulation of axon‐related genes and the increased presence of myelinated fiber innervation, we conducted morphological observations. TEM revealed synapse‐like structures formed between axonal terminals and VSMCs in both plaque‐bearing and non‐plaque‐bearing vessels (**Figure** **3**A,B; Figure S3A–F, Supporting Information). Quantitative analysis showed significantly higher vesicle counts in both the immediate vicinity (within 100 nm) (Figure 3C) and more distal areas (beyond 100 nm) (Figure 3D) of the presynaptic membrane in plaque‐bearing vessels compared to non‐plaque‐bearing vessels. This finding suggests enhanced vesicle release and storage activity in plaque‐bearing vessels. Additionally, the length of the active zone was significantly greater in plaque‐bearing vessels (Figure 3E), indicating an increase in neural signaling or communication, possibly reflecting adaptations in neurovascular interactions or remodeling processes.

Differential gene expression analysis revealed upregulation of genes involved in synaptic rearrangement, synaptic transmission, and synaptic integration in SCs from plaque‐bearing vessels (Figure 3F), indicating enhanced intercellular communication at the neurovascular interface. GSEA further showed that key pathways related to synapse assembly and synaptic structure or activity were upregulated in SCs from plaque‐bearing vessels (Figure S2E, Supporting Information). Meanwhile, expression of postsynaptic integration genes HOMER1, HOMER3, and NLGN1 was elevated in VSMCs from plaque‐bearing vessels (Figure 3G), supporting a role for synaptic activity in modulating neurovascular communication. To assess how myelinated nerve fibers innervate VSMCs, we performed triple IF staining. 3D confocal imaging revealed spatial co‐localization of axons (NF, green) and myelin sheaths (MPZ, purple) with VSMCs (SMA, red). We observed a significantly increased number of myelinated fibers innervating VSMCs in plaque‐bearing vessels (Figure 3H,J), suggesting enhanced neural innervation in these regions.

These findings indicate that the presence of vascular plaques is associated with increased neural signaling, suggesting that enhanced neurovascular communication may contribute to vascular remodeling and the progression of atherosclerosis.

### 2.4 Enhanced Interaction between SCs and VSMCs in Plaque‐bearing Vessels

**Figure 4:**
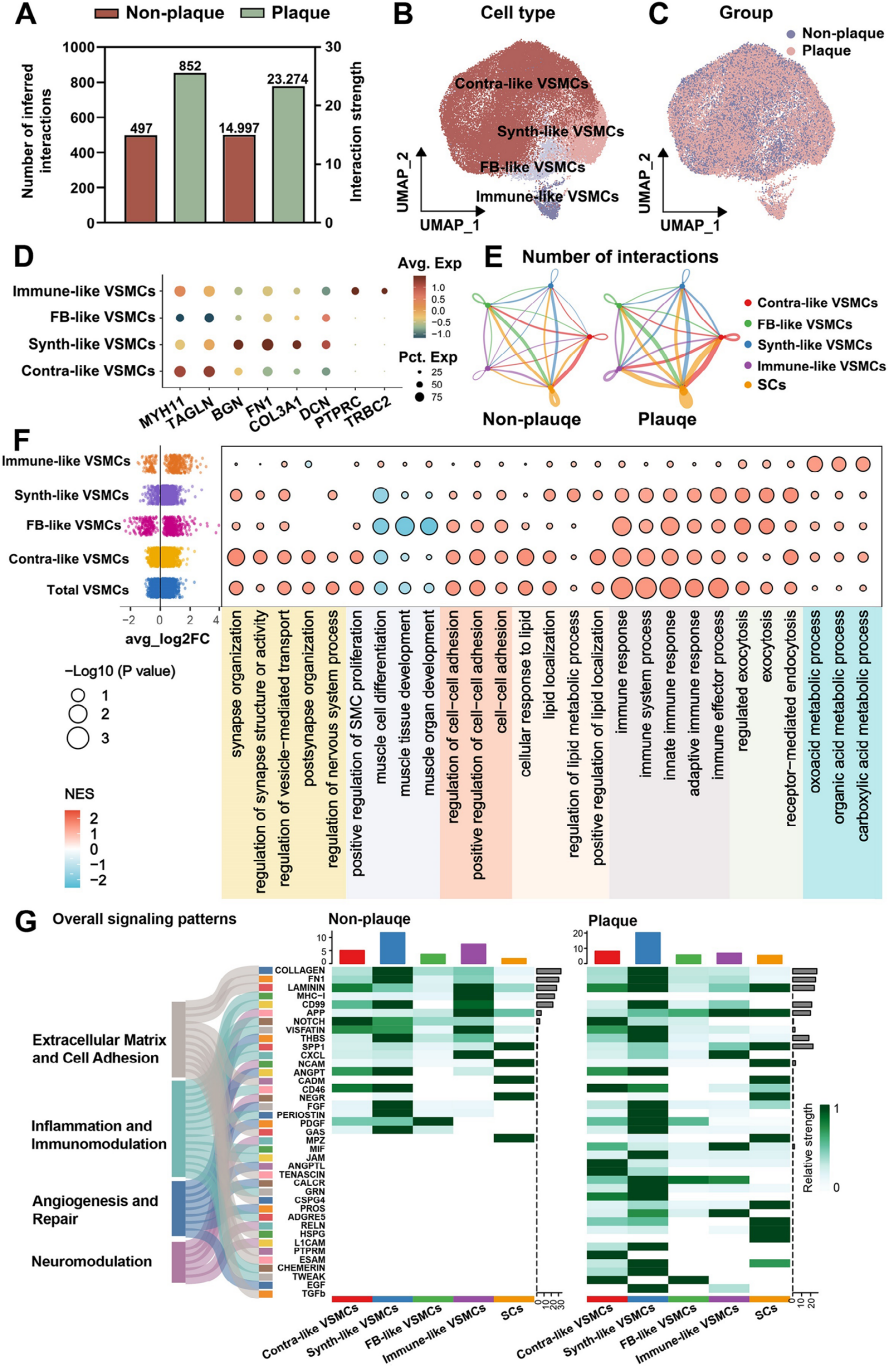
CellChat analysis of SCs and VSMCs interactions. A) Bar plots showing the number of inferred interactions and interaction strength between SCs and VSMCs in non‐plaque‐bearing and plaque‐bearing vessels. Plaque‐bearing vessels exhibited significantly higher interaction counts and interaction strength compared with non‐plaque‐bearing vessels. B) UMAP plot illustrating the four identified VSMC subtypes, including contractile‐like VSMCs (contra‐like VSMCs), synthetic‐like VSMCs (synth‐like VSMCs), fibroblast‐like VSMCs (FB‐like VSMCs), and immune‐like VSMCs. C) UMAP plot showing the distribution of cells in non‐plaque‐bearing and plaque‐bearing vessels. D) Dot plot showing the expression of key marker genes across VSMC subtypes. E) Network plots depicting the number of interactions among SCs and the four VSMC subtypes in non‐plaque‐bearing and plaque‐bearing vessels. SCs in plaque‐bearing vessels exhibited increased interactions. F) DEGs (left) analysis of VSMCs in plaque‐bearing versus non‐plaque‐bearing vessels. The plaque‐bearing vessels exhibited a significantly higher number of DEGs in four subtypes of VSMCs and total VSMCs. Gene set enrichment analysis (GSEA) (right) revealed subtype‐specific enrichment of biological pathways, including synaptic function, cell‐cell adhesion, lipid response, inflammation, and metabolic processes. G) Heatmap displaying overall signaling patterns in non‐plaque‐bearing and plaque‐bearing vessels. The plaque‐bearing vessels showed an upregulation of signaling pathways involved in extracellular matrix and cell adhesion, inflammation and immunomodulation, angiogenesis and repair, and neuromodulation, indicating that enhanced cellular communication may contribute to intracranial plaque progression. n = 8 donors.

We investigated interactions between SCs and other cell types and found that SCs exhibited the highest number of interactions with VSMCs (Figure S3G, Supporting Information). To further explore whether SCs contribute to ICAS progression by modulating VSMCs, we performed CellChat analysis. The results revealed an increase in both the number and strength of SC–VSMC interactions in plaque‐bearing vessels compared to non–plaque‐bearing vessels (**Figure** **4**A), suggesting a potential role for SCs in plaque progression. To assess the heterogeneity of VSMCs, we performed re‐clustering, which identified 15 distinct VSMC subclusters (Figure S3H, Supporting Information). Marker gene analysis confirmed that each subcluster expressed unique gene signatures (Figure S3I, Supporting Information). Based on these markers, we classified the 15 subclusters into four major VSMC subtypes: contractile‐like VSMCs (contra‐like), synthetic‐like VSMCs (synth‐like), fibroblast‐like VSMCs (FB‐like), and immune‐like VSMCs (Figure 4B). Each subtype exhibited a distinct gene expression profile consistent with its functional role (Figure 4D). The UMAP plot displayed the distribution of VSMC subtypes between plaque‐bearing and non–plaque‐bearing vessels (Figure 4C). DoubletFinder identified only 25 doublets among 38261 VSMCs (Figure S3K, Supporting Information), confirming that the vast majority of cells represented genuine biological populations. To explore the potential functional roles of the VSMC subtypes, enrichment analysis showed that functional modules such as synaptic activity, VSMC‐specific functions, cell adhesion, and immune response were differentially enriched across the four subtypes and the overall VSMC population (Figure 4F).

Cell interaction network analysis revealed that SCs interacted with all four VSMC subtypes in both plaque‐bearing and non–plaque‐bearing vessels, but the extent of these interactions was significantly greater in the plaque‐bearing group (Figure 4E). These interactions involved signaling pathways critical to vascular homeostasis and disease progression.

Specifically, SC–VSMC interactions were enriched for pathways involved in neuromodulation (APP, MPZ, RELN, L1CAM), extracellular matrix and cell adhesion (TENASCIN, CSPG4, HSPG, PTPRM, ESAM, and TGFb), inflammation and immunomodulation (MIF, JAM, GRN, ADGRE5, CHEMERIN, and TWEAK) and angiogenesis and repair (ANGPTL, CALCR, PROS, and EGF). Compared to non–plaque‐bearing vessels, plaque‐bearing vessels exhibited an overall increase in signaling activity (Figure 4G). Further analysis showed that both incoming and outgoing signaling between SCs and VSMCs were significantly elevated in plaque‐bearing vessels (Figure S4A,B, Supporting Information). Collectively, these findings indicate that SCs are active participants in the cellular dynamics of ICAS, particularly through enhanced interaction with VSMCs that may influence vascular remodeling and disease progression.

### 2.5 SPP1‐ITGB1 Signaling Pathway Potentially Regulates Intracranial Plaque Progression

**Figure 5:**
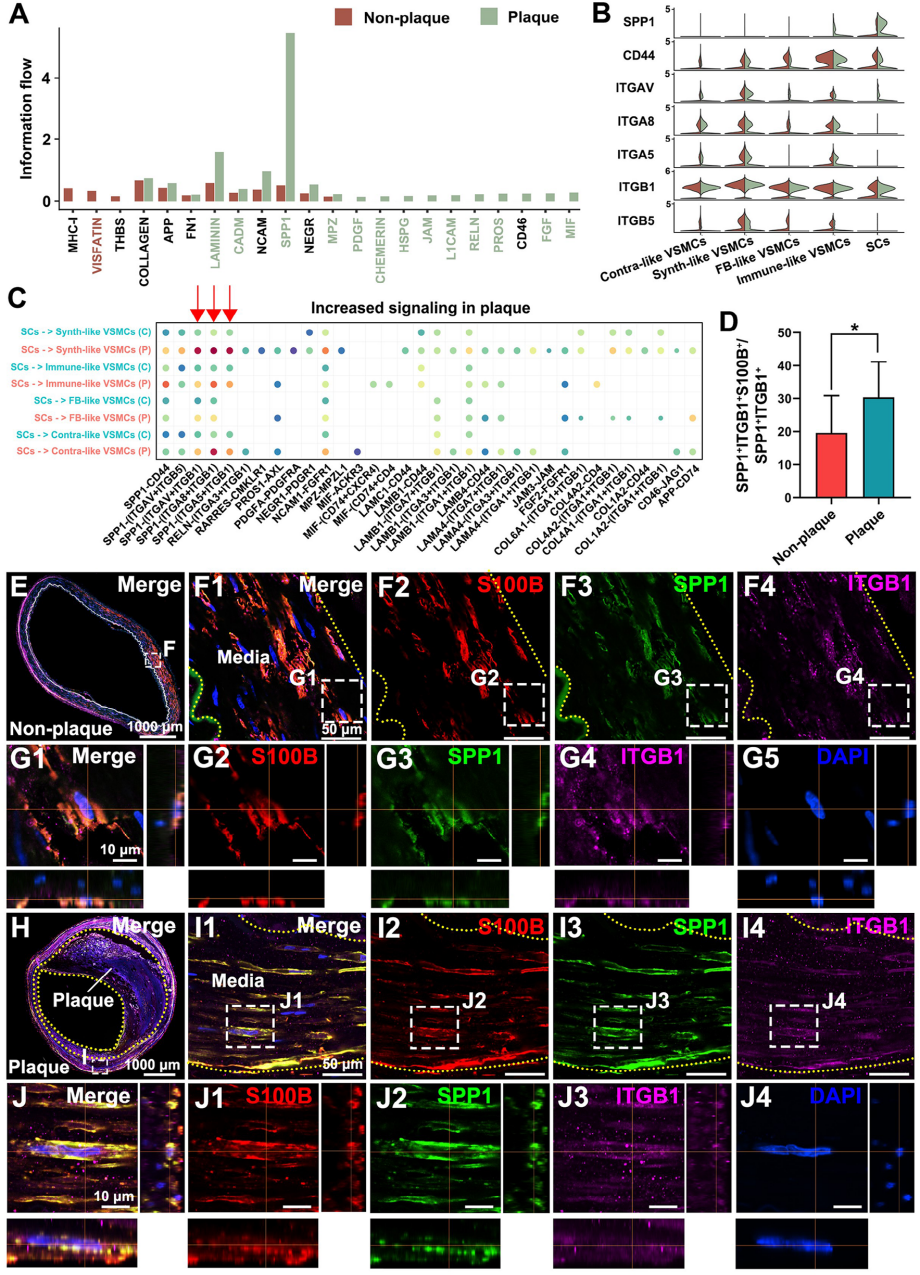
Enhanced communication between SCs and VSMCs in plaque‐bearing vessels. A) Information flow analysis between SCs and VSMCs revealed increased signaling in plaque‐bearing vessels compared to non‐plaque‐bearing vessels. B) Violin plots showing increased expression of key SPP1 signaling genes, including SPP1, CD44, ITGAV, ITGA8, ITGA5, ITGB1, and ITGB5, in plaque‐bearing vessels versus non‐plaque‐bearing vessels in different cell types (contra‐like VSMCs, synth‐like VSMCs, FB‐like VSMCs, immune‐like VSMCs, and SCs). C) Dot plot showing significantly upregulated signaling pathways in plaque regions, with SPP1 signaling being highly prominent. Pathways between SCs and different VSMC subpopulations are identified, with a focus on the SPP1‐ITGB1 axis. D) Quantitative analysis showed that the proportion of SPP1, ITGB1, and S100B positive cells in plaque‐bearing vessels was higher than that in non‐plaque‐bearing vessels. Data are presented as mean ± SD, * *P* < 0.05 (paired samples t‐test, n = 4 donors). E–G) IF staining in non‐plaque‐bearing vessels showed co‐localization of SPP1 (green) and ITGB1 (purple) within S100B‐positive SCs (red). H–J) IF staining in plaque‐bearing vessels revealed co‐localization of SPP1 and ITGB1 within SCs.

We evaluated the intensity of communication between SCs and VSMCs in both plaque‐bearing and non–plaque‐bearing vessels. Our results revealed that in plaque‐bearing vessels, signaling pathways involving SPP1, LAMININ, CADM, and MPZ were significantly enhanced (**Figure** **5**A; Figure S5A–D, Supporting Information). Comparative analysis showed that although all four pathways were upregulated in plaque‐bearing vessels, SPP1 exhibited the most prominent differential expression (Figure 5B), suggesting that these signaling molecules may play key roles in intercellular communication within atherosclerotic regions. Mapping these signaling pathways to their corresponding ligands and receptor markers revealed that the SPP1–ITGB1 signaling axis was particularly upregulated in plaque‐bearing vessels compared with non–plaque‐bearing vessels (Figure 5C). This result suggests a potential functional role for this pathway in the progression of intracranial atherosclerosis.

To validate the significance of the SPP1‐ITGB1 signaling axis, we performed IF staining on intracranial artery sections. The co‐localization of SPP1 and ITGB1 was observed within the SC population (S100B^+^) in both plaque‐bearing (Figure 5J) and non–plaque‐bearing vessels (Figure 5G). Quantitative analysis demonstrated that the density of SPP1 secreted by SCs and its receptor ITGB1 was significantly higher in plaque‐bearing vessels compared to non–plaque‐bearing vessels (Figure 5D). Additionally, analysis of the same specimens revealed that the expression of SPP1 and ITGB1 in VSMCs was also elevated in plaque‐bearing vessels (Figure S6A–G, Supporting Information). These findings suggest that SCs may communicate with VSMCs via the SPP1–ITGB1 signaling pathway, contributing to plaque progression

Collectively, these results indicate that active SC–VSMC communication, particularly through the SPP1–ITGB1 axis, may play a regulatory role in the progression of ICAS. The interaction between these two cell types within the plaque microenvironment may represent a key mechanism driving disease advancement and offers a potential therapeutic target for stabilizing or reversing plaque development in patients with ICAS.

## 3 Discussion

SCs have traditionally been regarded as defining components of the peripheral nervous system, where they myelinate axons and support neuronal function.^[^ ^13^, ^14^, ^15^, ^16^, ^17^ ^]^ However, the role of myelinated nerve fibers within intracranial arterial vessels has been scarcely investigated,^[^ ^18^ ^]^ leaving it unclear whether and how these fibers contribute to vascular regulation and function in this unique anatomical context. In this study, we employed a combination of TEM, IF, and scRNA‐seq to identify the presence of SCs in the walls of human intracranial arteries. The consistency of findings across multiple methodologies, along with the expression of biologically relevant markers, makes false positives unlikely. Further in situ validation using techniques such as spatial transcriptomics or RNAscope could provide additional confirmation and spatial resolution of SC localization within the vascular wall.

The exact origin of SCs in intracranial arterial walls remains uncertain. One possibility is that these cells share developmental pathways with oligodendrocytes, which are responsible for myelination in the central nervous system. Notably, during brain development, oligodendrocytes have been shown to accompany and regulate vascular formation.^[^ ^11^, ^12^ ^]^ SCs in arterial walls may represent a developmental variant or residual population of these oligodendrocyte‐like cells. Another hypothesis is that SCs in intracranial vessels may be linked to ganglionic cells in regions such as the basal forebrain, raphe nuclei, locus coeruleus, or ventral tegmental area, which could project myelinated fibers to vascular structures. This is supported by studies showing that certain central nervous system neurons can influence vascular function either directly or through nerve‐mediated modulation of VSMCs.^[^ ^19^ ^]^

In atherosclerotic lesions, the vasculature undergoes both structural and functional changes.^[^ ^5^, ^6^, ^7^, ^8^, ^9^, ^10^ ^]^ One of the key findings of this study is the increased presence of SCs and associated myelination in plaque‐bearing vessels compared to non–plaque‐bearing vessels. TEM revealed clear differences in SC distribution between these two vessel types, suggesting adaptive changes in SCs within the intracranial atherosclerotic environment. The upregulation of axon‐related genes in SCs from plaque‐bearing vessels points to a potential mechanistic link between SCs and neurovascular regulation. Axon guidance—a process involving the directional growth of axons through interactions with the extracellular matrix and cellular cues—is essential for the formation of functional neural circuits and the remodeling of tissue architecture.^[^ ^20^ ^]^ In the context of atherosclerosis, SCs may facilitate the extension of myelinated nerve fibers within the arterial wall, thereby influencing the behavior of target cells such as VSMCs. Moreover, we identified synapse‐like structures between axon terminals and VSMCs, suggesting a novel form of neurovascular communication within the arterial wall. This mechanism may resemble the synapse‐like connections observed between neurons and smooth muscle cell junctions in small arteries of the central nervous system.^[^ ^21^ ^]^ Such structures could serve as channels for rapid, targeted communication, allowing SCs to modulate VSMC activity in response to physiological changes such as altered blood flow or inflammation.

The SPP1 pathway is well known for its roles in inflammation,^[^ ^22^ ^]^ autoimmunity,^[^ ^23^ ^]^ cellular adhesion,^[^ ^24^, ^25^, ^26^, ^27^ ^]^ and tissue damage.^[^ ^28^ ^]^ A previous study demonstrated that SPP1^+^ macrophages contribute to coronary perivascular adipose tissue fibrosis via SPP1–CD44/integrin‐mediated activation of fibroadipogenic progenitors, directly linking perivascular inflammation to the progression of coronary atherosclerotic stenosis.^[^ ^29^ ^]^ In advanced, symptomatic atherosclerosis, SPP1 serves as a hallmark of pro‐inflammatory macrophage subsets and promotes plaque destabilization by regulating inflammatory signaling, ECM remodeling, and intercellular interactions.^[^ ^30^ ^]^ Additionally, SPP1 is highly expressed in VSMCs located in the core region of human carotid artery plaques, where it drives phenotypic switching and ECM remodeling, contributing to vascular calcification and potentially modulating inflammatory responses.^[^ ^31^ ^]^ In our study, the SPP1–ITGB1 signaling axis may function as a key mediator of SC–VSMC communication, particularly in plaque‐bearing intracranial vessels. These findings offer novel insight into the molecular mechanisms underlying plaque progression in intracranial atherosclerosis. However, translating this discovery into clinical applications will require further investigation into the precise role of the SPP1–ITGB1 axis in ICAS. Unfortunately, a major limitation is the current lack of reliable animal models that accurately replicate the pathological features of human intracranial atherosclerosis, which hindered direct validation of these mechanisms in this study.

In conclusion, our study provides unprecedented insights into the role of SCs in the pathogenesis of ICAS, challenging the traditional view that has historically excluded these cells from vascular pathology.^[^ ^32^, ^33^, ^34^, ^35^, ^36^ ^]^ By participating in neurovascular and cellular remodeling processes, SCs may contribute to plaque progression in a previously unrecognized manner. This finding introduces the novel concept that neural elements can influence vascular behavior during atherosclerosis progression, potentially through SC–VSMC interactions. A deeper understanding of these mechanisms may offer new therapeutic strategies to control plaque development and improve clinical outcomes for patients with ICAS.

## 4 Experimental Section

### Human Specimens Collection and Ethical Approval

This study utilized specimens from 16 human cadaver donors, aged 51 to 100 years, including eight males and eight females. Of these, specimens from four donors were used for TEM, four to IF, and eight for scRNA‐seq. The study was conducted in accordance with the ethical principles outlined in the Declaration of Helsinki and was approved by the Institutional Ethics Committee of the Institute of Basic Medical Sciences, Chinese Academy of Medical Sciences (Approval Numbers: 009–2014, 031–2017, 2 022 125). All cadaveric samples were obtained through legally authorized body donation programs, with written informed consent provided by the donors or their legal next of kin. The basic clinical characteristics of the 16 donors included in this study are summarized in Table S1 (Supporting Information) (△ represents the vascular location sampled from each donor for TEM analysis; represents the vascular location sampled for IF analysis; √ represents the vascular location used for scRNA‐seq analysis).

### TEM

To assess the presence of myelinated SCs in the walls of intracranial arteries, TEM was performed on arterial specimens obtained from four cadaveric donors through the National Human Brain Bank for Development and Function. Details of the specific arteries analyzed are provided in Table S1 (Supporting Information). Arterial samples were immediately fixed in 2.5% glutaraldehyde in 0.1 m phosphate buffer (pH 7.4) at 4 °C for 24 h. Specimens were then post‐fixed in 1% osmium tetroxide in the same buffer for 2 h at 4 °C. Samples were gradually dehydrated through a graded ethanol series (30%, 50%, 70%, 90%, and 100%) followed by treatment with propylene oxide. Dehydrated tissues were embedded in epoxy resin and polymerized at 60 °C for 48 h. Semi‐thin sections (1 µm) were cut and stained with toluidine blue to localize regions of interest for TEM analysis. In plaque‐bearing vessels, focus was on plaque‐border regions (the junction between plaque and adjacent normal tissue). For non–plaque‐bearing vessels, two random fields per section were analyzed, selected from areas with uniform structure and distribution. Ultra‐thin sections (60–80 nm) were prepared using an ultramicrotome and mounted on copper grids. These sections were stained with 2% uranyl acetate for 15 min, followed by lead citrate to enhance contrast. Images were acquired using a JEM‐1400 Plus Transmission Electron Microscope (JEOL, Japan). Three sections were analyzed per artery. Image‐Pro Plus software was used to quantify the number of myelin sheaths, myelin sheath thickness, vesicle counts within 100 nm and beyond 100 nm of the presynaptic membrane, and the length of the active zone per bouton. Raw data for each parameter were statistically analyzed using paired‐sample t‐tests to compare differences between plaque‐bearing and non–plaque‐bearing vessels.

### IF Staining

To investigate the presence of SCs and myelinated nerve fibers within the walls of intracranial arteries—and to examine their potential synapse‐like interactions with VSMCs, IF staining was performed on specimens from four cadaveric donors. Table S1 (Supporting Information) presents the basic clinical characteristics of these donors and details of the specific arteries analyzed, including the anterior cerebral artery (ACA), middle cerebral artery (MCA), posterior cerebral artery (PCA), and basilar artery (BA). All specimens were analyzed using a paired study design, comparing plaque‐bearing vessels and non–plaque‐bearing vessels from the same donor.

Fixed tissues were cryoprotected by immersion in a 30% sucrose solution at 4 °C until fully infiltrated. The cryoprotected specimens were then embedded in optimal cutting temperature (OCT) compound and sectioned at a thickness of 30 µm using a cryostat. Sections were washed three times with 0.01 m PBS, treated with 0.3% Triton X‐100 for 30 min and then blocked with 10% normal goat serum for 1 h at room temperature. Primary antibody incubation was performed for 48 h at 4 °C, with antibodies diluted in blocking buffer at their optimal concentrations. After thorough washing with PBS, sections were incubated with fluorophore‐conjugated secondary antibodies for 3 h at room temperature. Nuclei were counterstained with 4′,6‐diamidino‐2‐phenylindole (DAPI) for 10 min. Following a final PBS wash, sections were mounted using antifade mounting medium. Details of the antibodies used are provided in Table S2 (Supporting Information). Sections were imaged using a Nikon Spatial Array confocal microscope, and Image‐Pro Plus software was used for quantitative analysis of positively stained cells.

Sections were cut perpendicular to the vessel lumen. For serial sections of each intracranial artery, one section was sampled every ten sections, with a total of three sections analyzed per artery. Quantitative analyses included the number of myelinated SCs, the count of myelinated nerve fibers innervating VSMCs, and the extent of SPP1–ITGB1 co‐localization within SCs in both groups. Statistical comparisons were conducted using paired‐sample *t*‐tests. For non–plaque‐bearing vessels, two random regions per section were analyzed in areas with uniform structure and distribution. For plaque‐bearing vessels, regions were specifically selected at the interface between the plaque and adjacent normal vascular tissue. Additionally, sections were also cut parallel to the lumen. In this case, one section was sampled every three sections, with a total of three sections analyzed per artery. The number of myelinated nerve fibers was quantitatively assessed and compared between groups using paired‐sample *t*‐tests.

Human Intracranial Artery Tissue Collection and Cell Dissociation: Human intracranial arterial samples—including the ACA, MCA, PCA, and BA—were obtained from eight cadaveric donors. The experiment followed a self‐paired design: vessels with plaques were categorized as plaque‐bearing vessels, while those without plaques were classified as non–plaque‐bearing vessels. All collected intracranial arterial samples were processed for scRNA‐seq analysis.

Arterial tissues were harvested within four hours postmortem. One non‐plaque‐bearing sample failed sequencing quality control and was excluded from the analysis. The basic clinical characteristics of the eight donors included in this study are summarized in Table S1 (Supporting Information). Following collection, the samples were washed in ice‐cold PBS and dissociated using the Tissue Dissociation Kit C (SeekMate K01501‐50, SeeGene) according to the manufacturer's instructions. DNase I (Sigma, 9003‐98‐9) treatment was applied optionally, depending on the viscosity of the homogenate. Cell count and viability were assessed using a Fluorescence Cell Analyzer (Countstar Rigel S2) with AO/PI staining. The cells were washed with RPMI 1640 medium (Gibco 11 875 119) and resuspended at a concentration of 1×10^6^ cells mL^−1^ in RPMI 1640 supplemented with 2% fetal bovine serum (FBS; Gibco 10100147C).

### ScRNA‐seq Library Construction and Sequencing

Single‐cell RNA‐seq libraries were prepared using the SeekOne Digital Droplet Single Cell 3′ Library Preparation Kit (SeekGene, Catalog No. K00202).^[^ ^37^ ^]^ Briefly, an appropriate number of cells were mixed with reverse transcription reagents and added to the sample well of the SeekOne Chip S3. Barcoded Hydrogel Beads (BHBs) and partitioning oil were then dispensed into the corresponding wells on the chip. Emulsion droplets were generated, followed by reverse transcription at 42 °C for 90 min, and enzyme inactivation at 85 °C for 5 min. Subsequently, cDNA was purified from the broken emulsion and amplified by PCR. The amplified cDNA was then cleaned, fragmented, end‐repaired, A‐tailed, and ligated to sequencing adaptors. An indexed PCR was performed to amplify DNA fragments representing the 3′ poly(A) regions of expressed genes, incorporating both cell barcodes and unique molecular identifiers (UMIs). Final indexed sequencing libraries were purified using VAHTS DNA Clean Beads (Vazyme, N411‐01), quantified using a Qubit fluorometer (Thermo Fisher Scientific, Q33226), and analyzed for size distribution using a Bio‐Fragment Analyzer (Bioptic, Qsep400). Libraries were sequenced on the Illumina NovaSeq 6000 platform with a paired‐end 150 bp (PE150) read configuration.

### Generation of the ScRNA‐seq Data

Fastp was used to trim primer sequences and perform quality control on raw reads.^[^ ^38^ ^]^ Cleaned reads, obtained after trimming with specific parameters, were used in subsequent analyses.

The SeekSoul Tools pipeline was applied to process the cleaned reads and generate the transcript expression matrix. First, cell barcodes and UMI sequences were extracted based on a defined pattern specifying the location of the barcode, linker, and UMI within each read. Barcodes were corrected using a whitelist. The corrected barcodes, along with their corresponding UMIs, were inserted into the read headers. Next, reads were aligned to the reference genome (GRCh38) using STAR version 2.5.1b.^[^ ^39^ ^]^ Reads containing barcode and UMI information were then assigned to the transcriptome using the featureCounts function from the Subread package (v1.6.4).^[^ ^40^ ^]^ The parameters ‐s and ‐t in featureCounts were adjusted according to library chemistry and annotation type: ‐s 1 was used for 3′ chemistry and ‐s 2 for 5′ chemistry. The parameter ‐t exon was used to count only exonic reads, while ‐t transcript included both exonic and intronic reads. The fracOverlap parameter was set to 0.5, and other parameters were left at their default values. Finally, a raw UMI count matrix—similar to the raw_feature_bc_matrix output of Cell Ranger,^[^ ^41^ ^]^ —was generated, based on barcodes and transcripts. A cell‐calling algorithm, similar to those implemented in Cell Ranger and EmptyDrops^[^ ^42^ ^]^ was then applied to filter the raw UMI matrix, resulting in a *filtered_feature_bc_matrix* containing only high‐confidence cells.

Data quality control, preprocessing, and dimensionality reduction were performed using Seurat (v4.0.0, Satija Lab).^[^ ^43^ ^]^ During quality control, cells were excluded if they expressed fewer than 200 genes or more than 4000 genes, or if the percentage of mitochondrial or red blood cell gene expression exceeded 10%. The NormalizeData function was used to normalize expression matrices from atherosclerotic and non‐atherosclerotic vessels. Highly variable genes were identified using the FindVariableFeatures function, which selected the top 2000 genes based on dispersion. Principal component analysis (PCA) was conducted for linear dimensionality reduction, and the top 20 principal components were selected for clustering analysis. Expression data from different samples were integrated using the *FindIntegrationAnchors* and IntegrateData functions, employing a canonical correlation analysis (CCA)‐based method. Cell clustering was performed using the FindClusters function at a resolution of 1, utilizing the Leiden algorithm. To visualize the data, Uniform Manifold Approximation and Projection (UMAP) was applied, allowing high‐dimensional data to be projected into low‐dimensional space while preserving global data structure. This enabled nonlinear dimensionality reduction and visualization of cell populations.

### Cell Type Annotation

Cell clustering was first performed on all scRNA‐seq data using the UMAP algorithm. Differential expression analysis between cell clusters was conducted using the FindAllMarkers function to identify representative differentially expressed genes (DEGs). Clustering was performed at a resolution of 0.7 using 30 principal components (PCs). Cell type identification and annotation were conducted based on classic markers for different cell types from the literature and databases: SOX10, PLP1, S100B and MPZ for SCs, PECAM1 and SELE for endothelial cell, DCN and LUM for FB, ACTA2 and TAGLN for VSMC, CD79A and MS4A1 for B cell, S100A9, S100A8 and FCN1 for monocyte, CD68, CD86 and CD163 for macrophage, TOP2A, MKI67, CD68, CD86 and CD163 for proliferating macrophage, CD2, CD3D and TRAC for T cell, TOP2A, MKI67, CD2, CD3D and TRAC for proliferating T cell, PLP1, PMP22 and S100B for canonical SCs, MBP, PLP1, PMP22, S100B, SYN3 and EGFL8 for neural‐like SCs, MBP, PLP1, PMP22, S100B, ACAT2, TAGLN, MYH11 and CNN1 for VSMC‐like SCs, PLP1, PMP22, S100B, ACAT2, TAGLN, MYH11, CNN1, SYN3 for mixed SCs, MYH11 and TAGLN for contra‐like VSMCs, BCN and FN1 for synth‐like VSMCs, COL3A1 and DCN for FB‐like VSMCs, and MYH11, TAGLN, PTPRC and TRBC2 for immune‐like VSMCs.

### Differential Expression Analysis

To understand the differences of biological functions and molecular characteristics in cell clusters, the FindMarkers function was used to identify DEGs within SC clusters and VSMC clusters. For the comparison of plaque‐bearing vessels and non‐plaque‐bearing vessels, cell clusters were first subsetted, then FindMarkers functions was applied again by setting ‘ident’ parameter as two different sample groups. Gene expression with Benjamini‐Hochberg adjusted P‐values < 0.05 in Wilcoxon rank‐sum tests were considered as significantly different.

### Gene Set Enrichment Analysis (GSEA)

Gene Set Enrichment Analysis (GSEA): GSEA was performed to evaluate functional enrichment between plaque‐bearing and non–plaque‐bearing vessels, using ranked lists of DEGs and the clusterProfiler R package.^[^ ^44^ ^]^ Genes were pre‐ranked by log₂ fold change, and enrichment was assessed against Kyoto Encyclopedia of Genes and Genomes (KEGG) gene sets. The normalized enrichment score (NES) was calculated to account for gene set size differences, and statistical significance was determined using the false discovery rate (FDR). Enrichment results were visualized using the ggplot2, ggpubr, and GseaVis packages.

### Cell‐Cell Communication Analysis

To explore intercellular communication networks in the context of atherosclerosis, particularly between SCs and VSMCs, the *CellChat* package was used.^[^ ^45^ ^]^ Expression count data and cell group information were extracted from the Seurat object using the GetAssayData function. A CellChat object was created via the CreateCellChat function, with the human ligand–receptor database (CellChatDB.human) imported. Overexpressed ligands, receptors, and ligand–receptor pairs were identified and mapped onto the protein–protein interaction network. Communication probabilities were computed using the computeCommunProb function and filtered using filterCommunication. The aggregateNet function was then used to summarize communication probability and interaction count across signaling pathways. Visualizations of cell–cell communication networks were generated using bubble plots, heatmaps, and other built‐in graphical functions.

### Statistical Analysis

All data are presented as mean ± standard deviation (SD). GraphPad Prism (version 8.0) was used for generating statistical plots, and SPSS (version 22.0) was used for statistical analyses. The Shapiro–Wilk test was applied to assess the normality of data distributions, and Levene's test was used to evaluate the homogeneity of variance. Sample sizes (n) for each statistical test are provided in the corresponding figure legends and results sections. Paired‐sample t‐tests were used to compare differences between plaque‐bearing and non–plaque‐bearing vessels within individual donors. A *p*‐value <0.05 was considered statistically significant.

## Conflict of Interest

The authors declare no conflict of interest.

## Author Contributions

Z.W. and Y.Z.H. contributed equally to this work. W.X. and Z.W. conceived and designed the research. Z.W., W.Y., M.D., Z.C., N.W., and C.M. were responsible for the human intracranial arteries collection form the donors. Y.Z.H., Z.W., Y.X., and X.W. were responsible for bioinformatics data analysis. Z.W. was responsible for the morphology experiment. Z.W., Y.Z.H., Y.X., and W.X. wrote the manuscript and reviewed and edited the manuscript.

## Supporting information



Supporting Information

## Data Availability

The data that support the findings of this study are available on request from the corresponding author. The data are not publicly available due to privacy or ethical restrictions.
