# Supplementary material for: Identification of Schwann Cells in Human Intracranial Arteries: Potential Regulatory Role in Atherosclerotic Plaque Progression
Source: Adv Sci (Weinh). 2025 Jun 26;12(32):e03033. doi: 10.1002/advs.202503033 (PMC12407329; doi:10.1002/advs.202503033)
Supplement: Supplementary file 1 — Supporting Information [file ADVS-12-e03033-s001.docx]

**Supplemental publication material**


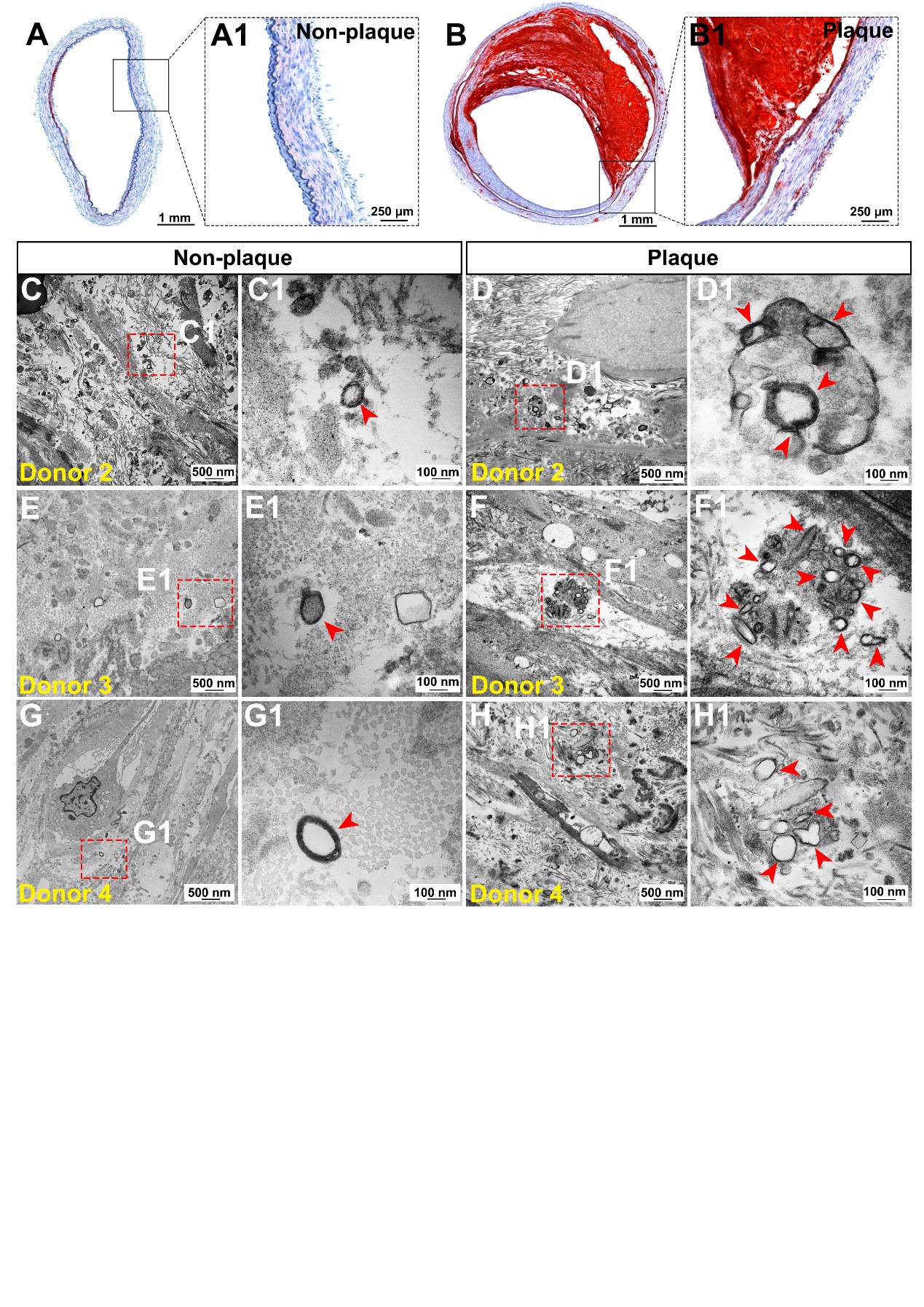


**Supplemental Figure S1.** Identification and characterization of SCs in human intracranial arterial walls. A, B) Representative images revealed prominent lipid deposition (red, Oil Red O staining) within plaque-bearing vessels, in contrast to negligible staining in non-plaque-bearing vessels (scale bar, 1 mm and 500 nm). n = 4 donors. C-H) TEM showed myelin sheaths in the intracranial artery across different donors analyzed in a self-paired experimental design. Plaque-bearing vessels showed increased clustering of myelin sheaths compared with non-plaque-bearing vessels (scale bar, 500 nm and 100 nm, n=4 donors). Red arrows indicated typical lamellar myelin sheath structures.

**
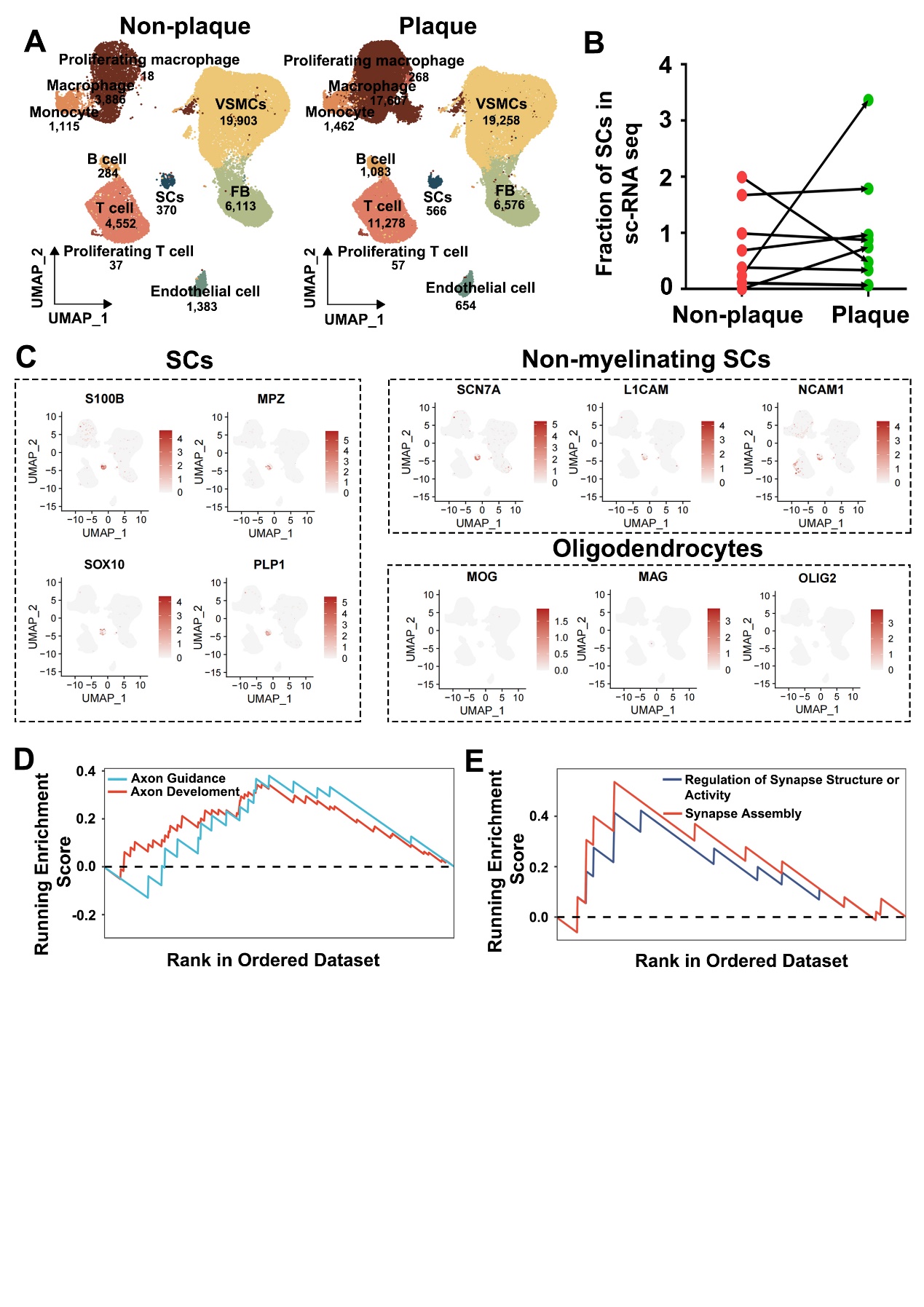
**

**Supplemental Figure S2.** ScRNA-seq revealed distinct features of SCs in plaque-bearing and non-plaque-bearing vessels. A) UMAP plots showed the distribution and number of various cell types in the two groups. The cell populations that have been identified include SCs, VSMCs, endothelial cell, FB, B cell, monocyte, T cell, proliferating T cell, macrophage, and proliferating macrophage. The number of each cell type was determined in plaque-bearing and non-plaque-bearing vessels, respectively. B) Fraction of SCs identified by scRNA-seq in non-plaque-bearing vessels and plaque-bearing vessels. Each pair of connected dots represented data from an individual donor. The red dots indicated non-plaque regions, and the green dots represented plaque regions. C) UMAP visualization of marker gene expression in SCs and oligodendrocytes.

SCs expressed S100B, MPZ, SOX10, and PLP1. Non-myelinating SCs expressed SCN7A, L1CAM, and NCAM1. No distinct oligodendrocyte clusters expressed MOG, MAG, and OLIG2. D) Gene set enrichment analysis (GSEA) results illustrated the upregulation of axon development (NES = 1.327, P = 0.097) and axon guidance (NES = 1.186, P = 0.269) pathways in SCs from plaque-bearing vessels compared to non-plaque-bearing vessels. E) GSEA showed upregulation of pathways related to the regulation of synapse structure or activity (NES = 1.565, P = 0.036) and synapse assembly in plaque-bearing vessels (NES = 1.557, P = 0.038).


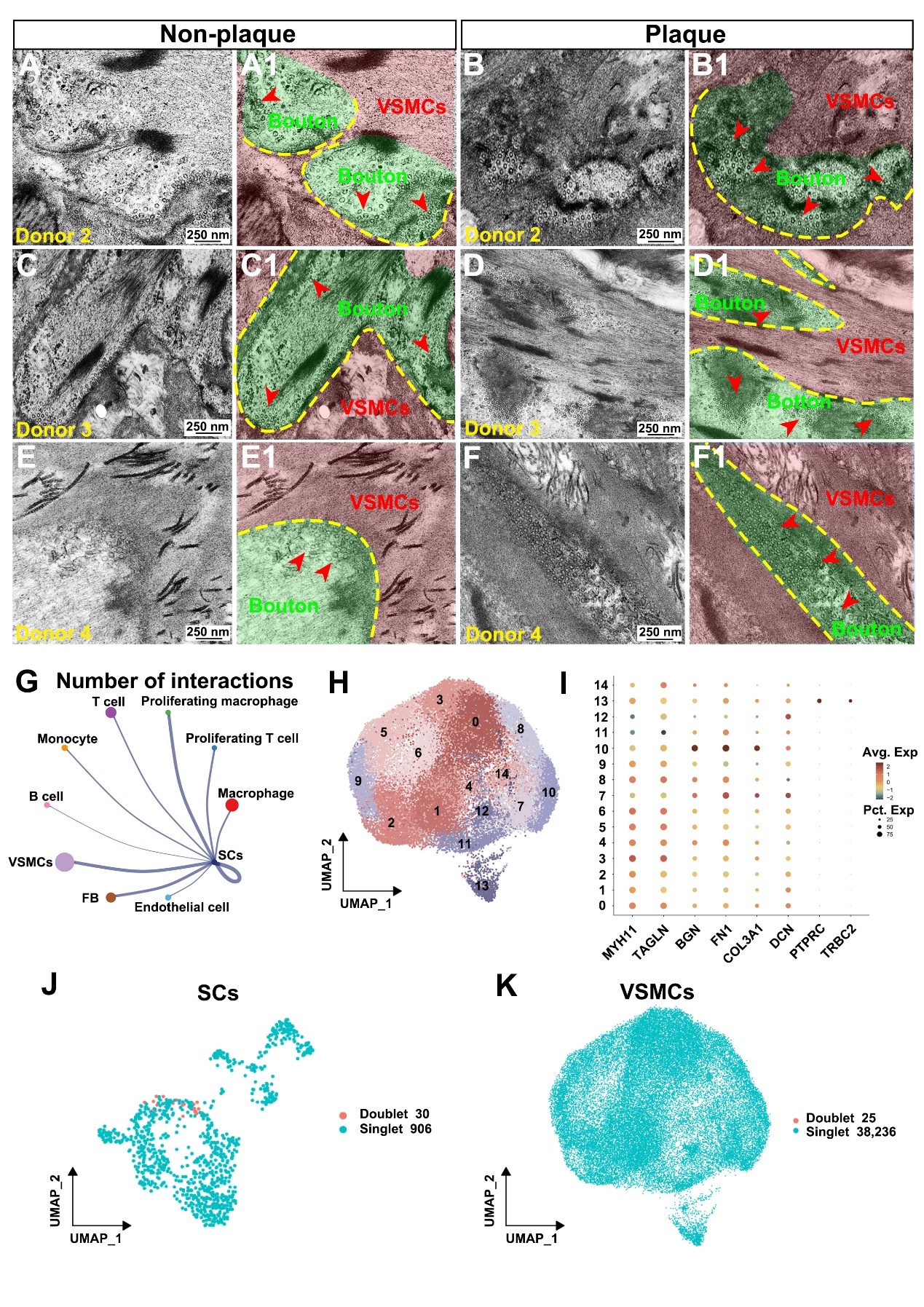


**Supplemental Figure S3.** Synaptic-like structures in human intracranial arteries. A-F) TEM showed synapse-like structures between axonal terminals (green) and VSMCs (red) in non-plaque-bearing vessels (A, C, and E) and plaque-bearing vessels (B, D, and F) of the intracranial artery in different donors (n = 4). Red arrowheads indicated presynaptic vesicles. Yellow-dotted line represented the synaptic active zone. Scale bars, 250 nm. G) Cell-cell interactions revealed a quantitative representation of interactions between SCs and other cell types, with the highest number of interactions observed between SCs and VSMCs. H) UMAP plot of re-clustered VSMCs, identified 15 distinct subclusters based on marker gene expression profiles. I) Dot plot of marker gene expression for the 15 VSMC subclusters. The color intensity represented the average gene expression level, and the dot size indicated the percentage of cells expressing each marker. J) DoubletFinder analysis of scRNA-seq data identifies SCs doublet contamination. The results showed that among 936 SCs, 30 doublets were detected. K) The results showed that among 38,261 VSMCs, 25 doublets were identified. n = 8 donors.


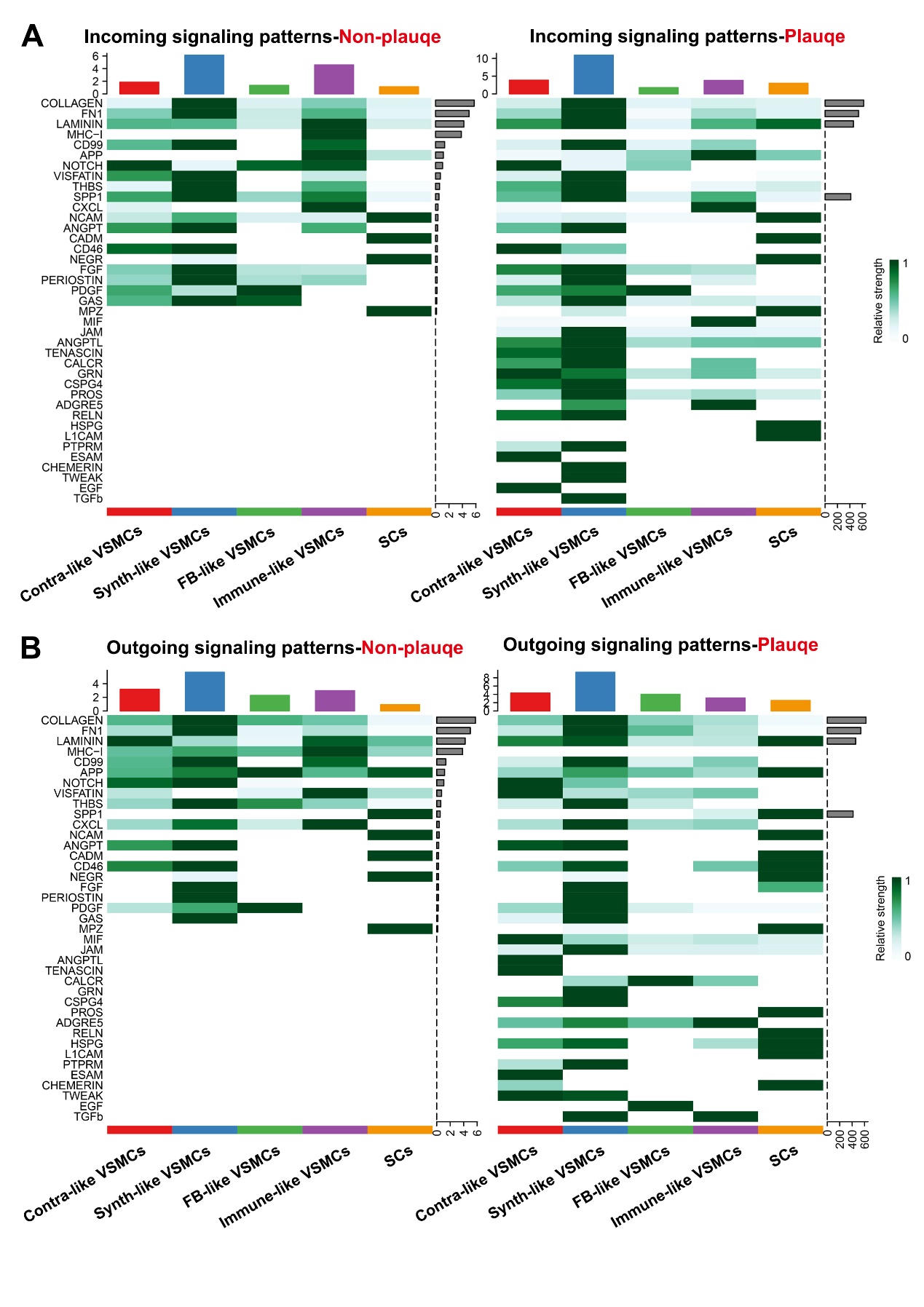


**Supplemental Figure S4.** Altered incoming and outgoing signaling patterns in plaque-bearing vs. non-plaque-bearing vessels. A) Heatmap of incoming signaling patterns in non-plaque-bearing vessels (left) and plaque-bearing vessels (right), showing the relative strength of signals received by contra-like VSMCs, synth-like VSMCs, FB-like VSMCs, immune-like VSMCs, and SCs. The signaling strength was notably higher in plaque-bearing vessels, indicating altered signal reception in these cell types during plaque progression. B) Heatmap of outgoing signaling patterns in non-plaque (left) and plaque-bearing vessels (right), demonstrating the strength of signals sent by VSMCs and SCs. Plaque-bearing vessels exhibited stronger outgoing signals, suggesting that these cell types have altered signal transmission profiles in the context of atherosclerosis. n = 8 donors.


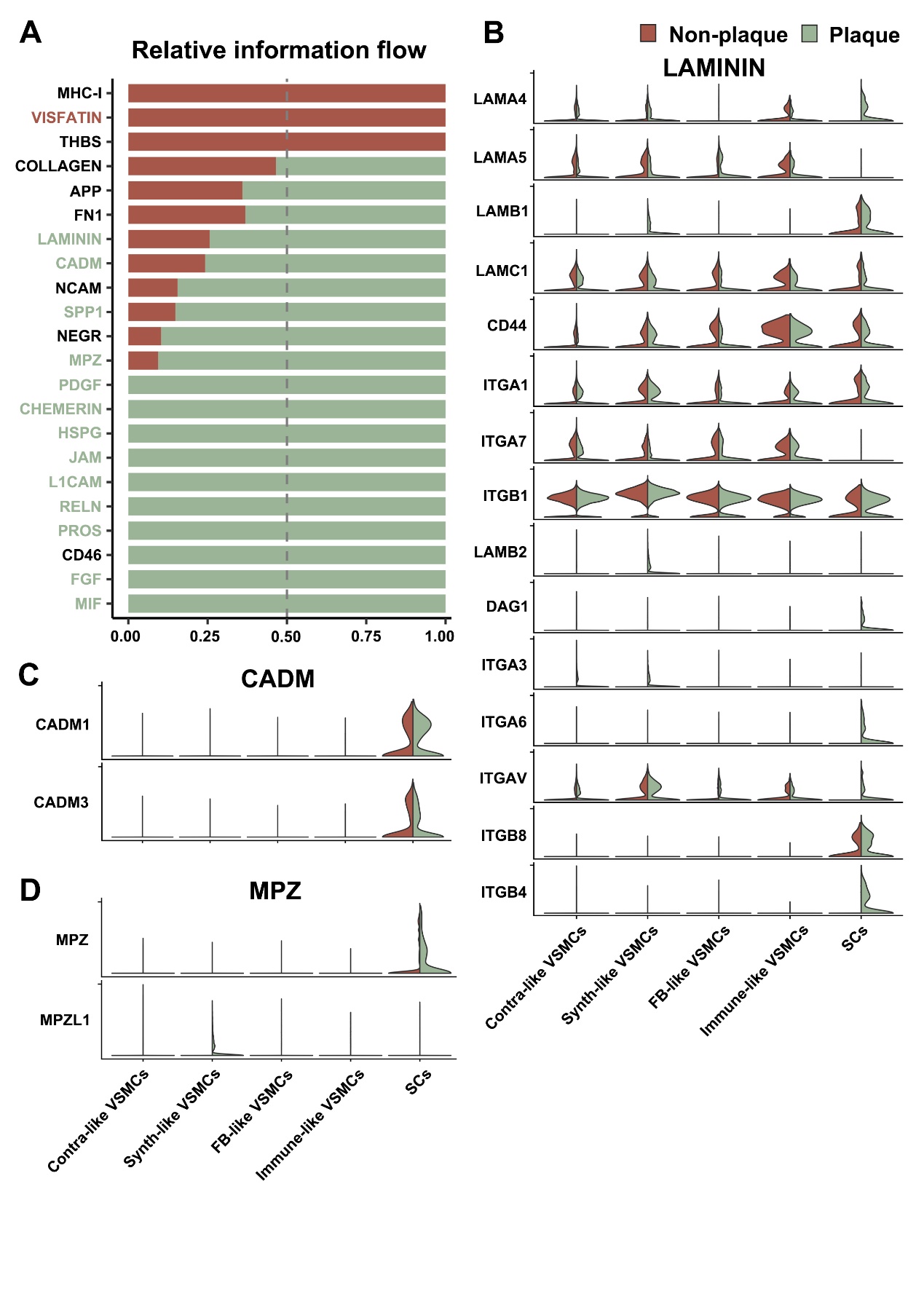


**Supplemental Figure S5.** Active communication between SCs and VSMCs in plaque-bearing vessels. A) Relative information flow between cell types in plaque-bearing vessels (green) and non-plaque-bearing vessels (red). B, C) Violin plots compared the expression of key extracellular matrix molecules (LAMININ, CADM) between plaque-bearing vessels (green) and non-plaque-bearing vessels(red). D) Violin plots compared the expression of key neuromodulation molecules between plaque-bearing vessels (green) and non-plaque-bearing vessels (red). n = 8 donors.


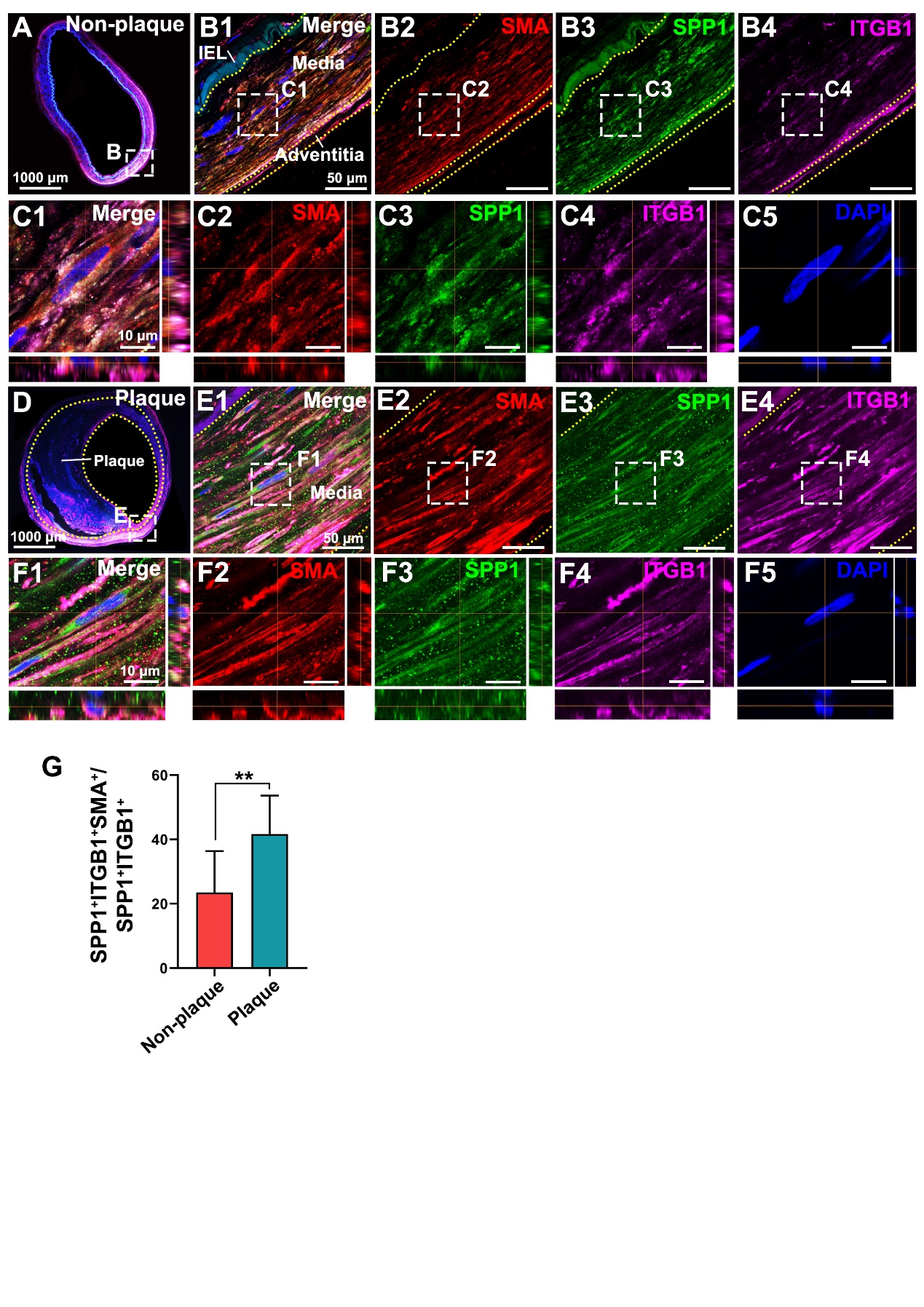


**Supplemental Figure S6.** Enhanced communication between SCs and VSMCs in plaque-bearing vessels. A-C) IF staining in non-plaque-bearing vessels showed co-localization of SPP1 (green) and ITGB1 (purple) within SMA-positive VSMCs (red). D-F) IF staining in plaque-bearing vessels revealed co-localization of SPP1 and ITGB1 within VSMCs. G) Quantitative analysis showed that the proportion of SPP1, ITGB1, and SMA-positive cells in plaque-bearing vessels was higher than that in non-plaque-bearing vessels. Data are presented as mean ± SD, ** *P* < 0.01 (paired samples t-test, n = 4 donors).

**Supplementary Table S1. The basic clinical characteristic of the cadaveric donors**

| **Donor ID** | **Age (years)** | **Gender** | **Cause of Death** | **Application** | **Anatomical origin of each donor** | | | |
| --- | --- | --- | --- | --- | --- | --- | --- | --- |
|  |  |  |  |  | **ACA** | **MCA** | **PCA** | **BA** |
| 1 | 87 | Female | Renal Failure | TEM |  |  |  |  |
| 2 | 89 | Female | Organ Failure | TEM | △ |  |  |  |
| 3 | 71 | Male | Lung Carcinoma | TEM |  |  | △ |  |
| 4 | 79 | Male | Ischemic Stroke | TEM |  |  |  | △ |
| 5 | 88 | Female | Vulvar Cancer | IF | ○ |  |  |  |
| 6 | 93 | Female | Heart Failure | IF |  |  | ○ |  |
| 7 | 87 | Male | Hepatocellular Carcinoma | IF |  | ○ |  |  |
| 8 | 93 | Male | Ischemic Stroke | IF |  |  |  | ○ |
| 9 | 86 | Female | Malignant Neoplasm | scRNA-seq | **√** | **√** | **√** | **√** |
| 10 | 100 | Female | Organ Failure | scRNA-seq | **√** | **√** | **√** | **√** |
| 11 | 83 | Female | Cholangiocarcinoma | scRNA-seq | **√** | **√** | **√** | **√** |
| 12 | 84 | Female | Sepsis | scRNA-seq | **√** | **√** | **√** | **√** |
| 13 | 79 | Male | Pancreatic Carcinoma | scRNA-seq | **√** | **√** | **√** | **√** |
| 14 | 51 | Male | Cardiogenic Shock | scRNA-seq | **√** | **√** | **√** | **√** |
| 15 | 95 | Male | Prostatic Carcinoma | scRNA-seq | **√** | **√** | **√** | **√** |
| 16 | 66 | Male | Intracerebral Hemorrhage | scRNA-seq | **√** | **√** | **√** | **√** |

**Supplementary Table S2. Primary and secondary antibodies used in the experiments.**

| **Antibodies** | **Product datasheet** | **Dilution** | **Source** |
| --- | --- | --- | --- |
| NF200 | Ab207176 | 1: 100 | Abcam |
| NF | MA1-2012 | 1: 200 | ThermoFisher |
| S100B | PA578161 | 1: 500 | ThermoFisher |
| S100B | ab218513 | 1: 100 | Abcam |
| MPZ | ab221364 | 1: 250 | Abcam |
| MPZ | Ab183868 | 1: 100 | Abcam |
| α-SMA | F3777 | 1: 200 | Sigma |
| SPP1 | 6696-RTM1-P0 | 1: 100 | ThermoFisher |
| SPP1 | Ab214050 | 1: 200 | Abcam |
| ITGB1 | 14-0299-82 | 1: 100 | ThermoFisher |
| ITGB1 | Ab30394 | 1: 200 | Abcam |
| Alexa Fluor 488 - AffiniPure Goat Anti-Rabbit | A-11070 | 1: 200 | Invitrogen |
| Goat Anti-Rabbit IgG H&L | ab150080 | 1: 200 | Abcam |
| Alexa Fluor 647 - AffiniPure Goat Anti-Rabbit | 110-605-046 | 1: 200 | Jackson Immunoresearch Laboratories |
| Goat Anti-Rabbit IgG H&L | ab150077 | 1: 200 | Abcam |
| Alexa Fluor 594 - AffiniPure Goat Anti-Mouse | 115-585-062 | 1: 200 | Jackson Immunoresearch Laboratories |
| Alexa Fluor 647 - AffiniPure Goat Anti-Mouse | ab150115 | 1: 200 | Abcam |
| Goat Anti-Rat IgG H&L | Ab175671 | 1: 500 | Abcam |
| DAPI | P36966 | — | ThermoFisher |
